# Cultural adaption and psychometric validation of the Danish Illness Identity Questionnaire (IIQ-DK) in adolescents and emerging adults with type 1 diabetes

**DOI:** 10.1016/j.heliyon.2022.e09109

**Published:** 2022-03-16

**Authors:** Marianne Vie Ingersgaard, Dan Grabowski, Kasper Olesen

**Affiliations:** a Steno Diabetes Center Copenhagen, Health Promotion Research, Borgmester Ib Juuls Vej 83, 2730, Herlev, Denmark; b National Institute of Public Health, Faculty of Health Sciences, University of Southern Denmark, Studiestræde 6, 1455, Copenhagen, Denmark

**Keywords:** Young adults, Emerging adults, Young people, Diabetes, Chronic illness, Health identity, Illness identity

## Abstract

**Purpose:**

The Illness Identity Questionnaire (IIQ) captures the ways in which individuals integrate chronic illness into their identity. The objectives were to linguistically validate and culturally adapt a Danish language version of the IIQ, and to evaluate the psychometric properties of this Danish version.

**Methods:**

IIQ was adapted through a forward-backward translation process, content validity assessment, and cognitive interviews (n = 5). Data for psychometric analysis were collected through an online Danish version of the IIQ (IIQ-DK). Data quality, internal consistency, and item correlations were assessed. Confirmatory factor analyses (CFA) were conducted.

**Results:**

Cognitive interviews resulted in re-wordings of two items. 1176 adolescents and emerging adults (15–26 years) with type 1 diabetes completed the IIQ-DK. Floor and ceiling effects were demonstrated for most items. Analysis showed good internal consistency of scales, as well as internal and discriminant item validity. CFA fit statistics after including correlated residuals were good for all scales. CFA showed acceptably high factor loadings for all items except one.

**Conclusion:**

Results demonstrated good reliability and psychometric properties of the IIQ-DK, which may be forwarded to use in research and clinical practice as a robust instrument to measure illness identity in adolescents and emerging adults with type 1 diabetes.

## 1 Introduction

Type 1 diabetes is one of the most prevalent chronic illnesses in adolescents and emerging adults (collectively “young people”). The management of diabetes constitute a significant part of young people's daily lives, involving blood-glucose monitoring, insulin therapy administration, and regulations of physical activity and dietary carbohydrate intake, all of which are to be balanced to keep optimal glycaemic levels and avoid acute and long-term complications. Key developmental processes of identity formation take place during adolescence and emerging adulthood [1, 2, 3]. Chronic illness, such as diabetes, is known to affect individuals' sense of self and identity, imposing identity changes and challenges [4, 5, 6, 7, 8, 9, 10, 11, 12, 13]. Further, identity constitutes an important mechanism in how individuals manage their illness [4, 5, 6, 7, 8, 9, 10, 11, 12, 13]. It may either support or hinder an individual's management behaviours, depending on how the illness is integrated into the identity [14, 15]. Integrating diabetes into the identity in adaptive ways may thus be an important task during adolescence and emerging adulthood [14, 16].

While the concept of identity has gained increasing prominence in qualitative research, quantitative assessment of identity has been somewhat neglected in the literature. This may partly be due to a lack of validated questionnaires available to measure identity in relation to chronic illness. Only recently, the Illness Identity Questionnaire (IIQ) was introduced, designed to capture the different ways in which individuals integrate their chronic illness into their identity, i.e. their ‘illness identity’ [14]. In a factorial validity analysis, Oris et al. (2016) demonstrated acceptable psychometric properties for the IIQ and its ability to measure and differentiate between four dimensions of illness identity in young people with type 1 diabetes: rejection, acceptance, engulfment and enrichment [14].

‘Rejection’ refers to the degree to which a chronic illness is rejected as part of one's identity; hence, the illness is perceived as a threat or unacceptable to the self [14, 17, 18, 19, 20, 21]. ‘Acceptance’ is defined as the degree to which one accepts the chronic illness as part of one's identity, in addition to, but not at the expense of, other self-defining assets, and without feeling overwhelmed by the chronic illness [14, 17, 18, 19, 20, 21]. ‘Engulfment’ refers to the degree to which the illness dominates one's identity. People who are engulfed by their chronic illness define themselves entirely in terms of it, and at the expense of other self-assets [14, 17, 18, 19, 20, 21]. Finally, ‘enrichment’ is defined as the degree to which the chronic illness enriches one's sense of self and enables one to grow as a person. In these cases, the chronic illness has changed one's values, resulting in positive life changes [14, 17, 18, 19, 20, 21].

Based on data collected through the IIQ, the importance of illness identity to treatment adherence, glycaemic levels, and psychological well-being in young people with type 1 diabetes has been demonstrated [14]. However, knowledge regarding illness identity in young people, and the potential for illness identity to support or hinder diabetes management, remains novel, and such findings need to be confirmed by further evidence in other settings and cultures. To provide such evidence, research needs a well-validated instrument to assess illness identity. To date, however, the IIQ has only been validated in Dutch [14, 17] and English [22], and applied in different chronic illness populations in Belgium [14, 17, 18, 19, 20], Germany [23], and the US [22]. To be regarded as a robust instrument for measuring illness identity in research, and for making comparisons across settings, the IIQ needs further psychometric validation in other languages and cultures. Therefore, the aims of the present study were to translate and linguistically validate a Danish version of the IIQ that is conceptually equivalent to the original IIQ, and to assess the psychometric properties of the Danish version (IIQ-DK) in a sample of young people with type 1 diabetes. A valid tool for capturing illness identity may be important in research aimed at understanding how identity issues affect diabetes management and outcomes, and further to help young people overcome identity challenges.

## 2 Methods

### 2.1 Design

A translation and cultural adaption of the IIQ was performed. This was followed by a survey study among a national sample of young people (age 15–25 years) with type 1 diabetes in Denmark, to evaluate the psychometric properties of the IIQ-DK.

### 2.2 Linguistic and cultural validation of the IIQ-DK

The IIQ was translated into Danish using a forward-backward translation procedure [24, 25] by four researchers from a specialist diabetes hospital and research institution in Denmark. Three of the translating researchers were native speakers of Danish language and had excellent English language skills, while one researcher was a native English speaker with good mastery of Danish. Two independent forward translations were performed and compared. Backward translations were then undertaken by two other researchers. Inconsistencies in the wordings of items in the forward and backward translations were discussed until consensus was gained. The translation process was documented by MVI and reviewed by the translators.

To assess content validity, two Danish experts (DG and KO) assessed whether the items in the IIQ-DK draft version represented a relevant and comprehensive sample of the content domains of illness identity in a way that was culturally appropriate for young people in a Danish context. Both experts represented a social science perspective and were experienced in research on psychosocial aspects of diabetes care. One expert (DG) was experienced in identity research and theory in a health context.

The level of comprehensibility of the IIQ-DK draft was then tested in cognitive interviews [26] with five young people with type 1 diabetes (3/5 females; age range 16–26 years, mean age: 23 years). Participants completed the preliminary version of the IIQ-DK, and then underwent individual, face-to-face cognitive interviews using verbal probing techniques to assess the relevance and intelligibility of the items, the response alternatives, and the survey instructions [26]. Rewording of items and survey instructions was done based on consistent feedback from participants, if semantically or theoretically justified.

### 2.3 Data collection

Data for psychometric analysis were collected through an online questionnaire. The study population was identified through the Danish National Patient Registry (NPR). Individuals were eligible for inclusion if they were between 15-25 years of age, had type 1 diabetes, and had a Danish civil registration number. All eligible individuals (N = 3673) were invited via a postal invitation to participate in the online survey using a secure digital mailbox system. Data collection was conducted over a 6-week period from March to April 2020. One digital reminder was sent to non-responders after approximately two weeks.

### 2.4 Measures

The online questionnaire included the IIQ-DK version that had been through the cross-cultural adaption process. The IIQ-DK comprised a 5-item rejection scale, a 5-item acceptance scale, an 8-item engulfment scale, and a 7-item enrichment scale. All items were rated on a 5-point Likert scale that ranged from “strongly disagree” (1 point) to “strongly agree” (5 points). A sum-score for each scale was calculated for each respondent. Higher scale scores indicated higher levels of rejection, engulfment, acceptance, and enrichment, respectively. Wordings of items in the IIQ-DK are provided in Supplementary Table 1.

### 2.5 Psychometric validation methods

Data quality was evaluated by mean and median scale scores. Floor and ceiling effects were assessed for each item; if more than 15% of respondents achieved the lowest or highest score, floor or ceiling effects were present, respectively [27]. To assess internal consistency of the IIQ-DK, Cronbach's alphas (α) and average inter-item correlations were calculated for the four scales individually. An α level between 0.70 and 0.95 was deemed acceptable [28, 29]. An average inter-item correlation of minimum 0.30 was deemed good; a high average inter-item correlation (above 0.80) was perceived as an indication of redundancy, while a correlation near 0 indicated a non-meaningful construct [30]. Further, it was assessed whether each item had a higher correlation with the sum-score of the rest of the items in its own scale (i.e., internal item convergence) than with those of the other scales (i.e., discriminant item validity) [31, 32]. Internal item convergence was considered satisfactory if an item correlation was ≥0.40. Acceptable discriminant validity was supported when a correlation between an item and its own scale was higher than its correlation with the other scales [33].

Confirmatory factor analyses (CFA) were conducted to evaluate the factorial validity and reliability of the scales in the IIQ-DK. Given that the four IIQ-DK scales were specified a priori, it was tested whether a one-factor solution for each scale individually provided a valid fit for the data. Maximum likelihood mean variance (MLMV) was used as estimation method. The MLMV option produces a mean and variance adjusted Chi-square test of model fit, which accounts for the non-normal distribution of the data. To identity the model, the factor loading was fixed at 1.0 for the first item in each respective scale. Estimate of the variance for the items explained by the latent factor (R^2^) and standardized factor loadings were estimated; a cut-off of ≥0.40 was used to identify acceptable factor loadings [34]. Initially, the CFA model was fitted with no correlated residuals allowed. To optimize model fit, we then allowed a minimal number of modifications, if theoretically justified. The Chi-square index (${\text{χ}}^{2}$) was used to assess the discrepancy between the sample and fitted covariance matrix; an insignificant test indicated good fit [35]. However, the ${\text{χ}}^{2}$ is extremely sensitive to sample size, and in large samples it tends to result in a rejection of the model. Therefore, less sensitive model fit indices were additionally assessed. These include Goodness-of-Fit index (GFI) > 0.90 [36], Comparative Fit Index (CFI) and Tucker Lewis Index (TLI) > 0.95 for good fit, and 0.9–0.95 for acceptable fit [35, 37], Root Mean Square Error of Approximation (RMSEA) < 0.05 for a well-fitting model, and <0.08 for acceptable fit [38], and Standardized Root Mean Square Residual (SRMR) < 0.05 for good model fit, and 0.05–0.08 for acceptable fit [39]. Correlations between latent factors of the four scales were calculated to assess discriminant validity; inter-factor correlations of >0.80 were considered to indicate a lack of discriminant validity [34]. CFA was conducted using IBM SPSS AMOS 25.

### 2.6 Ethics approval

The study was registered at and complies with the personal data protection policy in the Capital Region of Denmark (journal no. P-2020-255) and was conducted in line with the principles of the Declaration of Helsinki. According to the Regional Committees on Health Research Ethics for The Capital Region of Denmark, the study does not require ethical approval, as it does not implicate the use of human biological materials (journal no. 20014509).

Interviewees gave their written informed consent to participate in the cognitive interviews. In the invitation to take part in the survey, participants were informed about the purpose of the study and informed that by completing and submitting the survey they provided consent to use the data for analysis and publication of the results. They were also informed that no personally identifiable data would be disclosed. The authors affirm that participants provided informed consent for publication of the study results.

## 3 Results

### 3.1 Cultural and linguistic validation

Upon reviewing the forward-backward translation report, the translators made no additional item adjustments, and agreed on all item translations. The result was an IIQ-DK draft, which was subject to face validity assessment. The experts found that the items matched the content of the English items and reflected the theoretical constructs embodied in each illness identity domain. The draft version was then taken into cognitive interviews, which revealed that participants understood most of the items and were able to respond to them by the response alternatives. However, the procedure led to modification of two items. All participants had reservations about item 10, perceiving the wording to be “provoking” (interviewee statement). To accommodate this, item 10 was reworded; ‘begrænsninger’ [‘limitations’] was reworded to ‘ulemper’ [‘disadvantages’], and ‘pålægger’ [‘imposed’] was reworded to ‘fører med sig’ [‘brings’]. Further, participants had difficulties comprehending and responding to item 9 which was adapted to fit Danish linguistics culture; from ‘Jeg er i stand til at passe diabetes ind i mit liv’ [I am able to place diabetes in my life] to ‘Jeg har lært at leve med min diabetes’ [I have learned to live with my diabetes]. The outcome was the IIQ-DK which was considered final and used in the survey.

### 3.2 Sample characteristics

A total of 1176 young people with type 1 diabetes completed the survey, representing a response rate of 32%. Six responses were deleted due to missing data on age and gender, resulting in a final sample size of 1170 respondents. Detailed participant characteristics are provided in Table 1. 60.60% of the respondents were women. The mean age was 20.43 years (range 15–26 years). Mean diabetes duration was 9.73 years (range 0–25 years). Age and diabetes duration were equally distributed among respondents. The sample represented young people from all five regions in Denmark.

**Table 1:** Sample characteristics.

	Descriptive statistics
**Gender, female (%)**	709 (60.60)
**Age, mean years (SD), range**	20.43 (3.09), 15-26
**Clinical parameters (SD), range**	
Diabetes duration in years	9.73 (5.20), 0-25
Insulin administration (pump)	702 (60.00)

**Region, n (%)**	
Capital Region of Denmark	285 (24.36)
Region Zealand	166 (14.19)
Southern Region of Denmark	265 (22.65)
Central Region of Denmark	306 (26.15)
North Denmark Region	148 (12.65)

**Occupation, n (%)**	
Students	820 (70.09)
Working	251 (21.45)
Not working∗	99 (8.56)

**Level of education (ongoing or completed), n (%)**∗∗	
Elementary school	215 (18.41)
Secondary education (High school or equivalent)	433 (37.07)
Secondary education (Vocational)	159 (13.61)
Lower tertiary education (2–3 years)	36 (3.08)
Medium tertiary education (3–4 years)	155 (13.27)
Higher tertiary education (more than 4 years)	170 (14.55)

Legend: Descriptive statistics are given as frequency (percent) for categorical variables and mean (standard deviation) for continuous variables. ∗ = Homemakers, unemployed, on maternity or sick leave, or early retirement. ∗∗ = Missing values: n = 12 (i.e. not reported).

### 3.3 Data quality analysis

Item-level descriptive statistics are shown in Supplementary Table 1. The means (SD) of the total scale scores were 12.26 (4.35) for the rejection scale, 18.70 (4.09) for the acceptance scale, 21.48 (6.68) for the engulfment scale, and 21.69 (5.75) for the enrichment scale (Table 2). All items had no missing data. Item analysis showed that responses to the five response alternatives were evenly distributed for five of the items (median = 3 for items 2, 4, 22 and 24). Eight items were right-skewed with a median of 4 (item 6–10, 19, 21, and 23), and 12 were left-skewed with a median of 2 (item 1, 3, 5, 11–18, and 20) (Supplementary Table 1). Floor or ceiling effects were demonstrated for 20 items, as >15% of the respondents chose the “strongly disagree” or the “strongly agree” alternative.

**Table 2:** IIQ-DK scale scores.

Scale	Mean total score	SD	Observed/possible values		% at lowest score	% at highest score
			Lowest	Highest		
Rejection	12.26	4.35	5/5	25/25	5.21	0.34
Acceptance	18.70	4.09	6/5	25/25	0.51	7.78
Engulfment	21.48	6.68	8/8	40/40	4.62	0.34
Enrichment	21.69	5.75	7/7	35/35	0.68	1.11

There was good internal consistency for the rejection (α = 0.79), acceptance (α = 0.82), engulfment (α = 0.89), and enrichment (α = 0.88) scales (Table 3). The average inter-item correlation ranged from 0.44 to 0.51 for the scales (Table 3). Item-rest correlations (internal item convergence) were strong (>0.40) for all items (range 0.48–0.74) except item 7. All items except item 1 had stronger correlations with their own scale than with other scales (Table 3).

**Table 3:** Correlations between items and (i) the rest of the items in its own scale (item correlation), and (ii) the other scales.

	Item correlations with the sum-score of the rest of the items in own scale	Rejection scale	Acceptance scale	Engulfment scale	Enrichment scale
**Rejection items** (α = 0.79; average inter-item correlation = 0.44)					
Item 1	0.53∗∗∗	-	-0.62∗∗∗	0.37∗∗∗	-0.26∗∗∗
Item 2	0.67∗∗∗	-	-0.56∗∗∗	0.34∗∗∗	-0.30∗∗∗
Item 3	0.52∗∗∗	-	-0.38∗∗∗	0.22∗∗∗	-0.22∗∗∗
Item 4	0.50∗∗∗	-	-0.42∗∗∗	0.36∗∗∗	-0.17∗∗∗
Item 5	0.62∗∗∗	-	-0.50∗∗∗	0.27∗∗∗	-0.30∗∗∗

**Acceptance items** (α = 0.82; average inter-item correlation = 0.48)					
Item 6	0.60∗∗∗	-0.50∗∗∗	-	-0.19∗∗∗	0.35∗∗∗
Item 7	0.49∗∗∗	-0.45∗∗∗	-	-0.09∗∗	0.35∗∗∗
Item 8	0.70∗∗∗	-0.61∗∗∗	-	-0.46∗∗∗	0.28∗∗∗
Item 9	0.66∗∗∗	-0.55∗∗∗	-	-0.50∗∗∗	0.30∗∗∗
Item 10	0.60∗∗∗	-0.46∗∗∗	-	-0.47∗∗∗	0.26∗∗∗

**Engulfment items** (α = 0.89; average inter-item correlation = 0.51)					
Item 11	0.70∗∗∗	0.33∗∗∗	-0.37∗∗∗	-	-0.08∗∗
Item 12	0.63∗∗∗	0.28∗∗∗	-0.24∗∗∗	-	0.03
Item 13	0.54∗∗∗	0.14∗∗∗	-0.20∗∗∗	-	0.03
Item 14	0.69∗∗∗	0.32∗∗∗	-0.35∗∗∗	-	0.01
Item 15	0.74∗∗∗	0.36∗∗∗	-0.38∗∗∗	-	-0.03
Item 16	0.73∗∗∗	0.32∗∗∗	-0.36∗∗∗	-	-0.05
Item 17	0.67∗∗∗	0.37∗∗∗	-0.40∗∗∗	-	-0.13∗∗∗
Item 18	0.69∗∗∗	0.37∗∗∗	-0.41∗∗∗	-	-0.12∗∗∗

**Enrichment items** (α = 0.88; average inter-item correlation = 0.50)					
Item 19	0.60∗∗∗	-0.29∗∗∗	0.33∗∗∗	-0.05	-
Item 20	0.64∗∗∗	-0.20∗∗∗	0.28∗∗∗	-0.02	-
Item 21	0.69∗∗∗	-0.33∗∗∗	0.37∗∗∗	-0.17∗∗∗	-
Item 22	0.72∗∗∗	-0.20∗∗∗	0.27∗∗∗	0.04	-
Item 23	0.69∗∗∗	-0.30∗∗∗	0.32∗∗∗	-0.03	-
Item 24	0.70∗∗∗	-0.27∗∗∗	0.33∗∗∗	-0.06∗	-
Item 25	0.65∗∗∗	-0.22∗∗∗	0.29∗∗∗	-0.02	-

∗ = *p* value ≤.05; ∗∗ = *p* value ≤.01; ∗∗∗*p* value = ≤ .001.

The rejection and engulfment scales had negative correlations with the acceptance (−0.67 and -0.44, respectively) and enrichment (−0.34 and -0.06, respectively) scales, but had a positive correlation with each other (0.42). The enrichment scale had a positive correlation with the acceptance scale (0.41), and a low negative correlation with the rejection (−0.34) and engulfment (−0.06) scales.

### 3.4 Confirmatory factor analysis

An unmodified model was initially assessed for each scale, however, model fit indices indicated poor fit to the data with most indices falling outside acceptable values in all four CFAs (unreported data). Subsequently, we performed modified CFA models on each scale, allowing correlations between a few residuals within factors justified by the similarity in wordings of items. The modified CFA model for the rejection scale (allowing correlation between residuals of item 3 and 5) provided a good model fit, with only ${\text{χ}}^{2}$ falling outside acceptable standards (*df* = 4; ${\text{χ}}^{2}$ = 21.470, *p* = 0.00; GFI = 0.993; CFI = 0.989; TLI = 0.973; RMSEA = 0.061; SRMR = 0.0181) (Supplementary Figure 1). Likewise, the modified CFA model for the acceptance scale (allowing correlation between residuals of item 6 and 7) provided an acceptable model fit (GFI = 0.986; CFI = 0.986; TLI = 0.966; RMSEA = 0.088; SRMR = 0.0294), however, ${\text{χ}}^{2}$ did not meet threshold values (*df* = 4, ${\text{χ}}^{2}$ = 40.266, *p* = .00) (Supplementary Figure 2). For the engulfment scale, the modified CFA model (allowing correlation between residuals of item 17 and 18) provided an acceptable model fit with only ${\text{χ}}^{2}$ falling outside acceptable standards (*df* = 19, ${\text{χ}}^{2}$ = 126.271, *p* = 0.00; GFI = 0.973; CFI = 0.979; TLI = 0.968; RMSEA = 0.069, SRMR = 0.0303) (Supplementary Figure 3). Finally, the modified CFA model for the enrichment scale also indicated an acceptable model fit with only ${\text{χ}}^{2}$ falling outside acceptable standards (*df* = 11, ${\text{χ}}^{2}$ = 81.777, *p* = .00; GFI = 0.981, CFI = 0.982; TLI = 0.965; RMSEA = 0.074; SRMR = 0.0265) (Supplementary Figure 4). All standardized factor loadings (Table 4) for the final CFA models were >0.4 (range 0.52–0.83 for the rejection scale, 0.36–0.87 for acceptance scale, 0.63–0.81 for engulfment scale, and 0.61–0.78 for engulfment scale), except for item 7 which was 0.36.

**Table 4:** Standardized factor loadings and R^2^ in modified CFA models.

Item	One-factor CFA models (scales)	
	Factor loading	R^2^ (%)
**Rejection scale**		
Item 1	0.61	37
Item 2	0.83	70
Item 3	0.53	28
Item 4	0.52	27
Item 5	0.75	56

**Acceptance scale**		
Item 6	0.47	22
Item 7	0.36	13
Item 8	0.81	66
Item 9	0.87	76
Item 10	0.77	60

**Engulfment scale**		
Item 11	0.75	56
Item 12	0.68	46
Item 13	0.60	36
Item 14	0.77	59
Item 15	0.81	66
Item 16	0.78	61
Item 17	0.63	40
Item 18	0.65	42

**Enrichment scale**		
Item 19	0.61	38
Item 20	0.65	42
Item 21	0.71	51
Item 22	0.74	54
Item 23	0.77	59
Item 24	0.78	61
Item 25	0.69	48

All inter-factor correlations were below 0.80; the inter-factor correlation was -0.76 between rejection and acceptance, 0.46 between rejection and engulfment, -0.39 between rejection and enrichment, -0.60 between acceptance and engulfment, 0.37 between acceptance and enrichment, and -0.03 between engulfment and enrichment.

As rejection and acceptance items were found to be highly inter-related, we performed an additional modified one-factor CFA model on combined rejection and acceptance items to test whether variances of these items could be explained by a common latent factor. The CFA allowed correlation between residuals of item 3 and 5, and between item 6 and 7. This model provided a poor model fit, with all model fit indices falling outside acceptable standards (*df* = 33; ${\text{χ}}^{2}$ = 700.11, *p* = 0.00; GFI = 0.875; CFI = 0.871; TLI = 0.824; RMSEA = 0.132; SRMR = 0.132) (Data not shown).

## 4 Discussion

This study is one of the first to report on the cultural, linguistical, and psychometric validation of the IIQ beyond its initial development, and the first to validate a Danish version of it. The series of psychometric tests of data demonstrate that the IIQ-DK possesses acceptable psychometric properties, and the reliability of the original IIQ is maintained. Results provide initial evidence for the utility of the IIQ-DK as a robust instrument suitable for young people with type 1 diabetes in Denmark. The empirical validation of the utility of the four scales in another language and setting attests to the distinct elements in each of the four illness identity constructs embedded in the IIQ.

The psychometric assessment of the IIQ-DK largely replicated the findings from previous validation studies of the IIQ showing similar internal consistency [14, 17, 19, 23]. The illness identity construct was prespecified to consist of four distinct dimensions (scales) with their own estimate of reliability. Study findings demonstrated clear internal consistency for each of these scale, comparable to results from other validation studies of the IIQ [14, 17, 19, 23]. The item correlation analysis revealed acceptable internal item convergence and discriminant validity; all items had a strong (≥0.40) correlation with the sum-score of the rest of the items in their own scale, and all items had a stronger correlation with their own scale than with the other scales (except for item 1), suggesting scale homogeneity. Item 1 had a stronger correlation with the acceptance scale (−0.62) than with its own scale (rejection) (0.53). This inter-item correlation is not optimal but reasonable. Rejection and acceptance items were highly inter-related; thus, these constructs can be argued to capture two opposites along one conceptual continuum. However, the results from the unidimensional CFA model on rejection and acceptance scales indicated poor model fit, suggesting that the variances of the items in the rejection and acceptance scales cannot be explained by the same latent factor. Thus, we argue that rejection and acceptance are not two opposites of one conceptual continuum, but rather two distinct concepts and should be kept as two separate scales in the IIQ-DK in line with the original scale. Results from the CFAs demonstrate the construct validity of the four scales. Providing acceptable model fit to the data, the four unidimensional CFA models and inter-factor correlations confirmed the existence of four distinct, but strongly inter-related, latent factors, each with items loading highly on its intended factor, which indicates scale homogeneity.

There were a few item residual correlations within the CFAs, which can result from either construct complexity (sub-constructs) or item redundancy [40]. The item residual correlations in this study are considered to relate to the rather similar semantics of the items as there seems to be a conceptual overlap in the item coverage of item 19 and 21 (‘Because of my diabetes, I have grown as a person’ and ‘Because of my diabetes, I have become a stronger person’), suggesting item redundancy. While there is some conceptual overlap between these items, the scale had a wide coverage of the measured construct, hence, the items with residual correlations were retained.

Every item loaded highly on its intended latent factor, however, one item (item 7) had a factor loading (0.36) slightly below the acceptable threshold, suggesting that the variance in the acceptance factor explained by this item is smaller compared to the rest of the items in the scale. While the factor loading for this item being slightly below threshold is not optimal, it is considered acceptable enough to recommend inclusion of the item in the scale for various reasons. The item had high face validity, contributed to the internal consistency of its scale indicated by the high Cronbach's α and average inter-item correlation, and it has been found to have optimal factor loadings in other validation studies [14, 17, 22, 23]. Further, the authors revaluated the translation of item 7, concluding that the item reflected a literal translation of the English phrasing of the item. As such, the acceptance scale in Danish can be considered acceptable.

Item-level descriptive statistics showed that all response alternatives were used for all items in each of the four scales, indicating no problem with the translation of items or response alternatives. However, the distribution of the item response alternatives was either highly right- or left-skewed for most of the items. It is generally argued that a good item have substantial variability and a symmetrical response distribution [32]. While this may be a good rule of thumb in many cases, we argue that this may not be suitable for the IIQ-DK items. The left-skewed response distribution for the items in the rejection and engulfment scales indicates that most young people neither reject their diabetes as part of their identity nor feel engulfed by it. Likewise, the right-skewed response distribution of the acceptance items indicates that most young people accept diabetes as part of their identity. Thus, rather than reflecting problems with the phrasing of items, the skewness of items may indicate that rejection and engulfment represent rather uncommon, but not non-occurring or irrelevant, illness identity dimensions.

The survey study had a relatively low response rate (32%). However, the study sample was rather large (n = 1176), and participants were sampled from a national cohort of all individuals with type 1 diabetes between the ages of 15–25 years in Denmark. Further, the sample represented a wide geographical sociodemographic variation in age, gender and educational background, which increases the generalizability of the study results. An analysis of non-responders was not possible to conduct due to missing data on non-responders.

### 4.1 Implications

The study findings may have significant implications for diabetes research and care for young people with type 1 diabetes. Considering the prominence of identity formation in adolescent and emerging adult development, identifying illness identity may be important to fully understanding and optimizing diabetes management, diabetes outcomes, and psychological well-being in this population. Current evidence suggest that, regarding diabetes outcomes, young people do indeed benefit when they accept or embrace their diabetes as a part of their identity, while the opposite is the case for young people who reject or feel engulfed by it [14]. Young people with type 1 diabetes who reject diabetes as part of their identity have been demonstrated to have poorer treatment adherence and higher glycaemic levels than do those who demonstrate more adaptive illness identities [14]. Similarly, high levels of engulfment have been found to relate to maladaptive psychological functioning and diabetes-related problems. On the contrary, high levels of acceptance have been demonstrated to be associated with good treatment adherence, fewer diabetes-related problems, and adaptive psychological functioning, and enrichment have been associated with better psychological functioning [14]. Such results call for efforts to promote adaptive illness identity development in young people with type 1 diabetes. The newly validated IIQ-DK can give rise to further investigations of associations between illness identity and diabetes-specific outcomes. Further, future research should apply the IIQ-DK to investigate the factors that determine illness identity development in young people with type 1 diabetes. These findings can then guide interventions and improvements in clinical diabetes practice.

## 5 Conclusion

This study validated the IIQ-DK, which has adequate reliability and validity. The IIQ-DK may further be used in research and clinical practice to understand illness identity in young people with type 1 diabetes. With the IIQ-DK, analyses can be conducted to examine the effects of illness identity on diabetes outcomes, and to examine the potential factors that determine illness identity development in young people with type 1 diabetes.

## Declarations

### Author contribution statement

Marianne Vie INGERSGAARD, Dan GRABOWSKI and Kasper OLESEN: Conceived and designed the experiments; Performed the experiments; Analyzed and interpreted the data; Contributed reagents, materials, analysis tools or data; Wrote the paper.

### Funding statement

This work was supported by a research grant from the 10.13039/100015223Danish Diabetes Academy, which is funded by the 10.13039/501100009708Novo Nordisk Foundation, grant number NNF17SA0031406.

### Data availability statement

The authors do not have permission to share data.

### Declaration of interests statement

The authors declare no conflict of interest.

### Additional information

No additional information is available for this paper.

## Appendix A Supplementary data

Supplementary material Supplementary material

IIQ-DK questionnaire _spl_in Danish_spl_ IIQ-DK questionnaire _spl_in Danish_spl_
