# Supplementary material for: Cultural adaption and psychometric validation of the Danish Illness Identity Questionnaire (IIQ-DK) in adolescents and emerging adults with type 1 diabetes
Source: Heliyon. 2022 Mar 16;8(3):e09109. doi: 10.1016/j.heliyon.2022.e09109 (PMC9280371; doi:10.1016/j.heliyon.2022.e09109)
Supplement: Supplementary material [file mmc1.docx]

**SUPPLEMENTARY MATERIAL**

**Article title:** Cultural adaption and psychometric validation of the Danish Illness Identity Questionnaire (IIQ-DK) in adolescents and emerging adults with type 1 diabetes

**Authors:** Marianne Vie Ingersgaard, Dan Grabowski, and Kasper Olesen

**Supplementary Table 1. Data quality of the IIQ-DK: item-level descriptive statistics, % endorsement of response options, and % missing data (n=1170)**

|  | **Mean (SD)** | **Median** | **Strongly disagree (%)** | **Disagree (%)** | **Neither agree or disagree (%)** | **Agree (%)** | **Strongly agree (%)** | **Missing (%)** |
| --- | --- | --- | --- | --- | --- | --- | --- | --- |
| **Item** 1. Jeg nægter at se min diabetes som en del af mig selv. (I refuse to see my diabetes as part of myself) | 2.15 | 2.0 | 32.99 | 35.04 | 19.32 | 9.06 | 3.59 | 0 |
| **Item 2.** Jeg vil helst ikke tænke på min diabetes. (I’d rather not think of my diabetes) | 2.86 | 3.0 | 17.35 | 25.98 | 20.60 | 25.90 | 10.17 | 0 |
| **Item 3.** Jeg taler aldrig med andre om min diabetes. (I never talk to others about my diabetes) | 2.12 | 2.0 | 32.31 | 39.83 | 14.62 | 9.7 | 3.68 | 0 |
| **Item 4**. Jeg hader, når andre siger noget til mig om min diabetes. (I hate being talked to about my diabetes) | 2.92 | 3.0 | 18.89 | 21.62 | 22.56 | 22.65 | 14.27 | 0 |
| **Item 5.** Jeg undgår bare at tænke på diabetes. (I just avoid thinking about my diabetes) | 2.21 | 2.0 | 32.39 | 33.25 | 18.46 | 12.65 | 3.25 | 0 |
| **Item 6.** Min diabetes hører ganske enkelt til mig som person. (My diabetes simply belongs to me as a person) | 3.66 | 4.0 | 4.19 | 11.54 | 22.48 | 38.12 | 23.68 | 0 |
| **Item 7.** Min diabetes er en del af, hvem jeg er. (My diabetes is part of who I am) | 3.47 | 4.0 | 6.67 | 14.02 | 23.93 | 36.15 | 19.23 | 0 |
| **Item 8.** Jeg accepterer, at jeg er en person med diabetes. (I accept being a person with diabetes) | 4.00 | 4.0 | 3.08 | 6.84 | 12.74 | 41.45 | 35.90 | 0 |
| **Item 9.** Jeg har lært at leve med min diabetes. (I have learned to live with my diabetes [original: I am able to place diabetes in my life]) | 3.95 | 4.0 | 2.91 | 7.69 | 14.96 | 40.26 | 34.19 | 0 |
| **Item 10.** Jeg har lært at acceptere de ulemper, som min diabetes fører med sig. (I have learned to accept the disadvantages [original: limitations] that my diabetes brings [original: imposed by my diabetes]) | 3.61 | 4.0 | 4.44 | 13.76 | 19.15 | 41.28 | 21.37 | 0 |
| **Item 11.** Min diabetes dominerer mit liv. (My diabetes dominates my life) | 2.56 | 2.0 | 16.84 | 35.13 | 26.92 | 16.92 | 4.19 | 0 |
| **Item 12.** Min diabetes har stærk indflydelse på, hvordan jeg opfatter mig selv. (My diabetes has a strong impact on how I see myself) | 2.47 | 2.0 | 21.88 | 35.64 | 20.51 | 17.69 | 4.27 | 0 |
| **Item 13.** Jeg er for optaget af min diabetes. (I am preoccupied with my diabetes) | 2.18 | 2.0 | 22.22 | 47.26 | 22.31 | 6.41 | 1.79 | 0 |
| **Item 14.** Min diabetes påvirker alle mine tanker og følelser. (My diabetes influences all my thoughts and feelings) | 2.34 | 2.0 | 27.86 | 31.97 | 22.56 | 13.41 | 4.19 | 0 |
| **Item 15.** Min diabetes opsluger mig fuldstændig. (My diabetes completely consumes me) | 1.83 | 2.0 | 45.90 | 34.10 | 13.08 | 5.13 | 1.79 | 0 |
| **Item 16.** Det er som om alt, hvad jeg gør, er påvirket af min diabetes. (It seems as if everything I do, is influenced by my diabetes) | 2.25 | 2.0 | 32.31 | 31.88 | 18.12 | 13.42 | 4.27 | 0 |
| **Item 17.** Min diabetes forhindrer mig i at gøre, hvad jeg virkelig har lyst til. (My diabetes prevents me from doing what I would really like to do) | 2.47 | 2.0 | 25.56 | 30.00 | 22.74 | 14.47 | 6.24 | 0 |
| **Item 18.** Min diabetes begrænser mig i mange ting, der er vigtige for mig. (My diabetes limits me in many things that are important to me) | 3.37 | 2.0 | 27.44 | 33.16 | 19.15 | 14.96 | 5.30 | 0 |
| **Item 19.** På grund af min diabetes er jeg vokset som person. (Because of my diabetes, I have grown as a person) | 3.55 | 4.0 | 2.99 | 11.20 | 28.74 | 40.00 | 16.07 | 0 |
| **Item 20.** På grund af min diabetes ved jeg, hvad jeg vil have ud af livet. (Because of my diabetes, I know what I want out of life) | 2.55 | 2.0 | 17.01 | 33.08 | 32.39 | 13.25 | 4.27 | 0 |
| **Item 21.** På grund af min diabetes er jeg blevet stærkere som person. (Because of my diabetes, I have become a stronger person) | 3.40 | 4.0 | 5.73 | 14.02 | 28.38 | 38.55 | 13.33 | 0 |
| **Item 22.** På grund af min diabetes har jeg indset, hvad der er virkelig vigtigt i livet. (Because of my diabetes, I realize what is really important in life) | 2.84 | 3.0 | 12.05 | 26.67 | 34.10 | 20.09 | 7.09 | 0 |
| **Item 23.** På grund af min diabetes har jeg lært en masse om mig selv. (Because of my diabetes, I have learned alot about myself) | 3.40 | 4.0 | 6.07 | 14.36 | 26.75 | 39.15 | 13.68 | 0 |
| **Item 24.** På grund af min diabetes har jeg lært at arbejde mig igennem problemer og ikke bare give op. (Because of my diabetes, I have learned to work through problems and not just give up) | 3.17 | 3.0 | 8.72 | 17.18 | 32.82 | 30.94 | 10.34 | 0 |
| **Item 25.** På grund af min diabetes har jeg lært at nyde nuet mere. (Because of my diabetes, I have learned to enjoy the moment more) | 2.79 | 3.0 | 13.76 | 24.96 | 36.67 | 18.12 | 6.50 | 0 |

**Supplementary Figure 1. Final CFA model for the rejection one-factor solution (standardized estimates)**

**
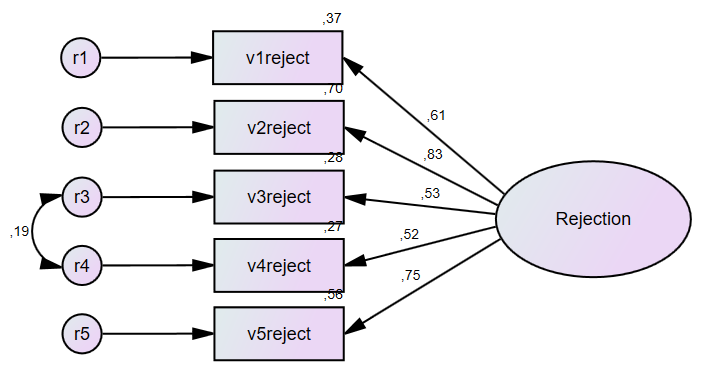
**

**Supplementary Figure 2. Final CFA model for the acceptance one-factor solution (standardized estimates)**

**
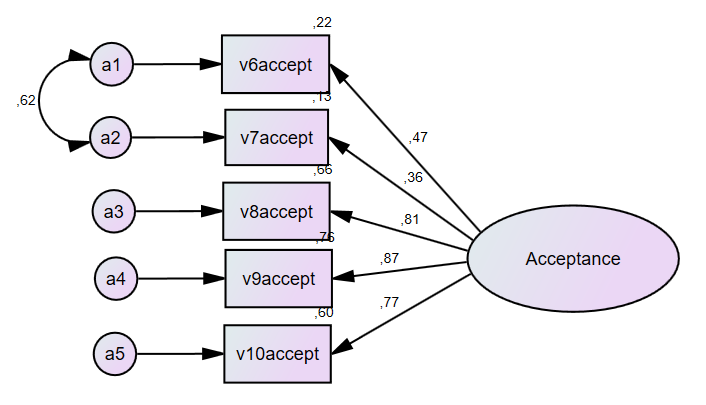
**

**Supplementary Figure 3. Final CFA model for the engulfment one-factor solution (standardized estimates)**

**
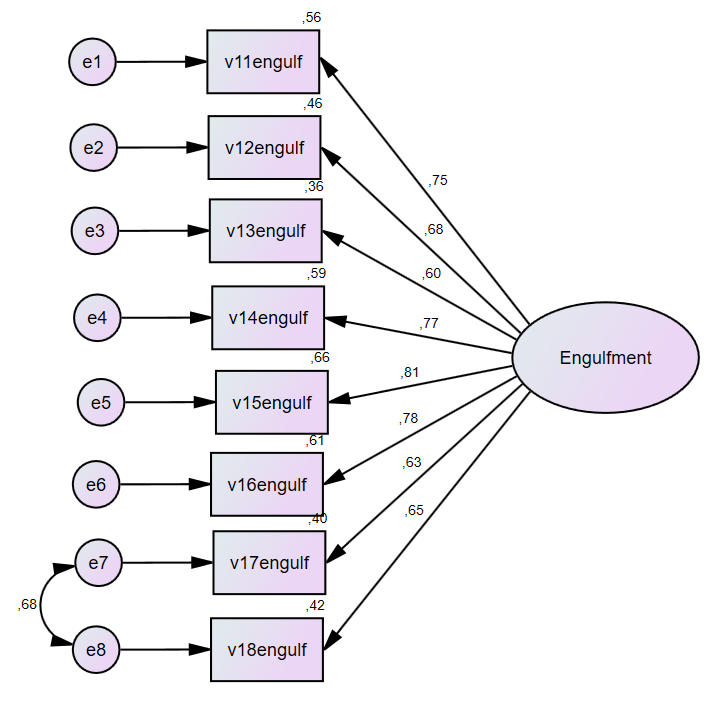
**

**Supplementary Figure 4. Final CFA model for the enrichment one-factor solution (standardized estimates)**

**
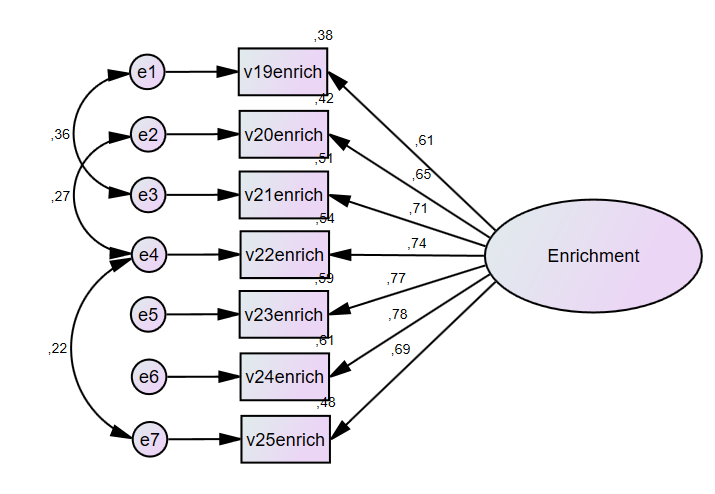
**
