# Supplementary material for: Cultural adaption and psychometric validation of the Danish Illness Identity Questionnaire (IIQ-DK) in adolescents and emerging adults with type 1 diabetes
Source: Heliyon. 2022 Mar 16;8(3):e09109. doi: 10.1016/j.heliyon.2022.e09109 (PMC9280371; doi:10.1016/j.heliyon.2022.e09109)
Supplement: IIQ-DK questionnaire _spl_in Danish_spl_ [file mmc2.docx]

|  | **Meget uenig (1)** | **Uenig (2)** | **Hverken uenig  eller enig (3)** | **Enig (4)** | **Meget enig (5)** |
| --- | --- | --- | --- | --- | --- |
| 1. Jeg nægter at se min diabetes som en del af mig selv. | ❑ (1) | ❑ (2) | ❑ (3) | ❑ (4) | ❑ (5) |
| 1. Jeg vil helst ikke tænke på min diabetes. | ❑ (1) | ❑ (2) | ❑ (3) | ❑ (4) | ❑ (5) |
| 1. Jeg taler aldrig med andre om min diabetes. | ❑ (1) | ❑ (2) | ❑ (3) | ❑ (4) | ❑ (5) |
| 1. Jeg hader, når andre siger noget til mig om min diabetes. | ❑ (1) | ❑ (2) | ❑ (3) | ❑ (4) | ❑ (5) |
| 1. Jeg undgår bare at tænke på diabetes. | ❑ (1) | ❑ (2) | ❑ (3) | ❑ (4) | ❑ (5) |
| 1. Min diabetes hører ganske enkelt til mig som person. | ❑ (1) | ❑ (2) | ❑ (3) | ❑ (4) | ❑ (5) |
| 1. Min diabetes er en del af, hvem jeg er. | ❑ (1) | ❑ (2) | ❑ (3) | ❑ (4) | ❑ (5) |
| 1. Jeg accepterer, at jeg er en person med diabetes. | ❑ (1) | ❑ (2) | ❑ (3) | ❑ (4) | ❑ (5) |
| 1. Jeg har lært at leve med min diabetes. | ❑ (1) | ❑ (2) | ❑ (3) | ❑ (4) | ❑ (5) |
| 1. Jeg har lært at acceptere de ulemper, som min diabetes fører med sig. | ❑ (1) | ❑ (2) | ❑ (3) | ❑ (4) | ❑ (5) |
| 1. Min diabetes dominerer mit liv. | ❑ (1) | ❑ (2) | ❑ (3) | ❑ (4) | ❑ (5) |
| 1. Min diabetes har stærk indflydelse på, hvordan jeg opfatter mig selv. | ❑ (1) | ❑ (2) | ❑ (3) | ❑ (4) | ❑ (5) |
| 1. Jeg er for optaget af min diabetes. | ❑ (1) | ❑ (2) | ❑ (3) | ❑ (4) | ❑ (5) |
| 1. Min diabetes påvirker alle mine tanker og følelser. | ❑ (1) | ❑ (2) | ❑ (3) | ❑ (4) | ❑ (5) |
| 1. Min diabetes opsluger mig fuldstændig. | ❑ (1) | ❑ (2) | ❑ (3) | ❑ (4) | ❑ (5) |
| 1. Det er som om alt, hvad jeg gør, er påvirket af min diabetes. | ❑ (1) | ❑ (2) | ❑ (3) | ❑ (4) | ❑ (5) |
| 1. Min diabetes forhindrer mig i at gøre, hvad jeg virkelig har lyst til. | ❑ (1) | ❑ (2) | ❑ (3) | ❑ (4) | ❑ (5) |
| 1. Min diabetes begrænser mig i mange ting, der er vigtige for mig. | ❑ (1) | ❑ (2) | ❑ (3) | ❑ (4) | ❑ (5) |
| 1. På grund af min diabetes er jeg vokset som person. | ❑ (1) | ❑ (2) | ❑ (3) | ❑ (4) | ❑ (5) |
| 1. På grund af min diabetes ved jeg, hvad jeg vil have ud af livet. | ❑ (1) | ❑ (2) | ❑ (3) | ❑ (4) | ❑ (5) |
| 1. På grund af min diabetes er jeg blevet stærkere som person. | ❑ (1) | ❑ (2) | ❑ (3) | ❑ (4) | ❑ (5) |
| 1. På grund af min diabetes har jeg indset, hvad der er virkelig vigtigt i livet. | ❑ (1) | ❑ (2) | ❑ (3) | ❑ (4) | ❑ (5) |
| 1. På grund af min diabetes har jeg lært en masse om mig selv. | ❑ (1) | ❑ (2) | ❑ (3) | ❑ (4) | ❑ (5) |
| 1. På grund af min diabetes har jeg lært at arbejde mig igennem problemer og ikke bare give op. | ❑ (1) | ❑ (2) | ❑ (3) | ❑ (4) | ❑ (5) |
| 1. På grund af min diabetes har jeg lært at nyde nuet mere. | ❑ (1) | ❑ (2) | ❑ (3) | ❑ (4) | ❑ (5) |
